# Challenges to the provision of diabetes care in first nations communities: results from a national survey of healthcare providers in Canada

**DOI:** 10.1186/1472-6963-11-283

**Published:** 2011-10-21

**Authors:** Onil K Bhattacharyya, Irit R Rasooly, Mariam Naqshbandi, Elizabeth A Estey, James Esler, Ellen Toth, Ann C Macaulay, Stewart B Harris

**Affiliations:** 1 Li Ka Shing Knowledge Institute, St. Michael's Hospital, 30 Bond Street, Toronto, ON M5B 1M8, Canada; 2 Centre for Studies in Family Medicine, Department of Family Medicine, Schulich School of Medicine and Dentistry, The University of Western Ontario, Suite 245-100 Collip Circle, London, ON N6G 4X8, Canada; 3 Department of Medicine, University of Alberta, 362 Heritage Research Medical Center, Edmonton, AB, T6G 2S2, Canada; 4 Participatory Research at McGill, Department of Family Medicine, McGill University, 515 Pine Avenue West Montreal, QC H2S 1W4, Canada

## Abstract

**Background:**

Aboriginal peoples globally, and First Nations peoples in Canada particularly, suffer from high rates of type 2 diabetes and related complications compared with the general population. Research into the unique barriers faced by healthcare providers working in on-reserve First Nations communities is essential for developing effective quality improvement strategies.

**Methods:**

In Phase I of this two-phased study, semi-structured interviews and focus groups were held with 24 healthcare providers in the Sioux Lookout Zone in north-western Ontario. A follow-up survey was conducted in Phase II as part of a larger project, the Canadian First Nations Diabetes Clinical Management and Epidemiologic (CIRCLE) study. The survey was completed with 244 healthcare providers in 19 First Nations communities in 7 Canadian provinces, representing three isolation levels (isolated, semi-isolated, non-isolated). Interviews, focus groups and survey questions all related to barriers to providing optimal diabetes care in First Nations communities.

**Results:**

the key factors emerging from interviews and focus group discussions were at the patient, provider, and systemic level. Survey results indicated that, across three isolation levels, healthcare providers' perceived patient factors as having the largest impact on diabetes care. However, physicians and nurses were more likely to rank patient factors as having a large impact on care than community health representatives (CHRs) and physicians were significantly less likely to rank patient-provider communication as having a large impact than CHRs.

**Conclusions:**

Addressing patient factors was considered the highest impact strategy for improving diabetes care. While this may reflect "patient blaming," it also suggests that self-management strategies may be well-suited for this context. Program planning should focus on training programs for CHRs, who provide a unique link between patients and clinical services. Research incorporating patient perspectives is needed to complete this picture and inform quality improvement initiatives.

## Background

Aboriginal people living in industrialized countries experience disproportionately high rates of type 2 diabetes mellitus (diabetes), diabetes complications, and associated risk factors when compared to their non-Aboriginal counterparts [1-4]. In Canada, Aboriginal peoples are comprised of three distinct Indigenous groups, namely First Nations, Inuit and Métis. Rates of diabetes are 2.5 to 4 times higher among First Nations people than the general population, with higher rates among women than men and a younger age at diagnosis [5]. In addition, some First Nations people have higher rates of documented smoking, obesity, hypertension and dyslipidemia and have increasing rates of serious diabetes-related complications [6-8]. This epidemiological trend requires urgent clinical action: primary prevention of diabetes is necessary to protect future generations, but initiatives to improve the quality of care provided to First Nations people already living with diabetes are urgently needed to reduce the risk of serious morbidities and mortality.

The term First Nations encompasses a diverse group of 615 communities, speaking upwards of 60 languages [9]. Together, these diverse nations make up the largest Aboriginal group in Canada, representing nearly 700,000 people of the total Aboriginal population (1.3 million) [10]. While a growing number of First Nations people are moving to urban and peri-urban areas, there are still currently over 600 recognized First Nations reserves in Canada, housing a population of approximately 470,000 [9]. The First Nations and Inuit Health Branch (FNIHB) of Health Canada is the primary provider of healthcare to First Nations communities. This is particularly true in remote and isolated areas, where provincial or territorial services are not readily available. While FNIHB provides most primary and emergency services on-reserve, the provinces and territories remain responsible for providing First Nations people with physician and hospital services. Recently, FNIHB has been working with communities to transfer the control of community-based health programs to the communities. As the transfer of services requires capacity on the ground, internal resources, and a governance system, transfer is being done on a community-by-community basis, with some communities opting out [11,12].

Improving quality of care requires translating evidence into clinical practice. A number of barriers such as knowledge, attitudes, and behavior have been shown to influence physician adherence to national clinical practice guidelines, which outline standards of care for diabetes [13]. It has been suggested that these barriers are more pronounced in Aboriginal contexts due to geographic isolation, cultural differences and the complex healthcare system described above [14-16]. Thus, an in-depth understanding of the barriers facing providers in First Nations settings is needed to bridge the gap between best evidence and current practice [17].

We conducted a mixed-methods two-phase study to bridge this gap. In the first phase, we qualitatively assessed the barriers, as perceived by healthcare providers, to providing optimal diabetes care as outlined by the Canadian Diabetes Association clinical practice guidelines [18]. In the second phase, we conducted a survey of 224 healthcare providers working in the 19 First Nations communities involved in the national Canadian First Nations Diabetes Clinical Management and Epidemiologic (CIRCLE) study to explore how healthcare providers rank barriers to diabetes care.

## Methods

### Phase I

A detailed description of methods used in the qualitative work conducted during the first phase of this study has been published elsewhere [19,20]. In summary, healthcare providers working in remote First Nations communities in the Sioux Lookout Zone in north-western Ontario, Canada were selected using snowball and criterion-based sampling to participate in focus groups or interviews about the barriers to diabetes care. The Sioux Lookout Zone is home to approximately 16,000 people in 28 isolated and semi-isolated Cree and Ojibway communities. Larger communities have nursing stations with two to four permanent nurse staff, while physicians visit three to ten days a month, depending on the community's size. Otherwise, physicians are based in the Zone Hospital in Sioux Lookout (population 5,000), where they take phone calls, review lab results, and have occasional patient-related teleconferences. Data collected from the interviews and focus groups were coded and thematically analyzed using NVIVO 2.0 software. All participants provided informed consent and ethics approval for this portion of the study was received from the Research Ethics Board (REB) at the University of Toronto, Health Canada, and the *Meno-Ya-Win *Hospital in Sioux Lookout.

### Phase II

The qualitative work in Phase I informed the development of a survey of healthcare providers as part of the CIRCLE study, a three-year national cross-sectional chart audit and survey study that documented the quality of diabetes care and rates of complications in 19 First Nations communities in Canada [6,7]. For the purposes of this paper, quality of care is defined as adhering to the treatment recommendations and targets outlined by the Canadian Diabetes Association 2008 Clinical Practice Guidelines [18]. High-quality care can help prevent diabetes-related complications [21] although it is recognized that both social determinants and genetics may also play an important role [22,23].

#### Survey Development

Three separate surveys were developed in October 2007 to target three different populations: (1) healthcare providers (physicians, nurses, dieticians/nutritionists, diabetes educators), (2) clinic managers, and (3) community healthcare representatives (CHRs). CHRs are lay health workers with variable training and experience that work with others in health care teams to improve and maintain the spiritual, physical, social and emotional well-being of individuals, families and their communities. One section of these surveys (*Improving Diabetes Care*) was based on the qualitative data and was common to all three surveys. The *Improving Diabetes Care *section of the survey asked healthcare professionals to rank their perception of the magnitude of impact (large, some, small, none and don't know) of fifteen different strategies for diabetes care. These fifteen strategies were drawn from the barriers identified in the previously described qualitative study. They were specifically re-phrased as strategies following the field-testing phase: representatives from participating communities were asked to review study materials and First Nations community members advocated for the more constructive language of "strategies to improve care" in order to shift the focus away from the more negative "barriers" that can be discouraging for communities and providers.

#### Survey Administration

One or two community research assistants were hired from each partnering community to carry out data collection for the CIRCLE study. All research assistants attended a three-day training session focused on privacy and confidentiality, informed consent, and the research protocol methodology. Research assistants developed a list of all healthcare providers working with patients diagnosed with diabetes in their community. These lists were developed in consultation with community healthcare facilities, health directors, the nurse-in-charge, and/or other relevant personnel. Research assistants contacted each healthcare provider on the list to obtain written informed consent. Consenting healthcare providers were supplied with the appropriate survey for completion. Surveys were either completed on the spot or were collected by the research assistants at a later date. Data collection was carried out from September 1 to November 30, 2008.

#### Study Population

The 19 partnering First Nations communities in the CIRCLE were recruited from seven provinces in Canada (British Columbia, Alberta, Saskatchewan, Manitoba, Ontario, Quebec, and Newfoundland and Labrador) and represented three isolation levels identified by Health Canada (isolated, semi-isolated and non-isolated) [24]. Clinic managers, a variety of healthcare workers (nurses, doctors, dieticians/nutritionists and diabetes educators), and CHRs were eligible to complete the survey if they were involved in diabetes care in one of the 19 partnering communities during the selected timeframe of September 1 to November 30, 2008. This three-month time frame was chosen so that the contribution of agency nurses and temporary replacements would not overshadow the core staff of the facilities/nursing stations.

#### Data Analysis

Survey data was input into SPSS version 17. A factor analysis was run to assess the number of factors underlying the survey questions. Descriptive analyses were performed to determine the frequency of responses ranked by providers for each of the impact categories (large, some, small, no impact and don't know) and to assess variation between communities. Pearson's chi-square test was used to test for significant differences between rankings of each survey item across isolation levels and healthcare provider type. Separate subgroup analyses were performed for isolation level and provider role to determine which levels differed significantly from one another. Each provider role was compared to every other provide role and each isolation level was compared to every other isolation level using separate analyses. Analyses were limited to responses of "large impact" in order to highlight those strategies perceived by providers as having the greatest impact on diabetes care. All chi-square tests were significant at the P < .05 level. Only significant results are presented in this paper.

#### Ethical Approval

Ethics approval for Phase II was received from the REB at The University of Western Ontario as the coordinating centre of the study and from the Health Canada REB on an annual basis as well as from the participating site academic institutions and the Chief and Council of the 19 participating communities. The participating communities are not identified to ensure anonymity and confidentiality outlined in the ethics agreements.

## Results

### Phase I

Twenty-four nurses, doctors, and community health representatives (CHRs) participated in qualitative interviews and focus groups. Detailed results arising from the interviews and focus groups have been previously published [19,20] and build on those demonstrated by Brown et al [25]. In summary, providers perceive barriers as falling into four categories: patient, provider, systemic, and environmental factors. Patient factors are the degree to which patients assume responsibility and control over their diabetes. Provider factors include barriers and facilitators to provider knowledge and skills, the implementation of clinical practice guidelines, and practice (re)organization. Lastly, systemic factors are defined as barriers and facilitators that relate to service accessibility and funding. An additional (non-modifiable) factor revealed from our study, and potentially distinct to Aboriginal care settings, was environmental factors as evident from the following quote - "*Where else do people not have running water, unpaved roads, no vegetables or they're three times the normal price?"*

The previous qualitative work was limited in that it did not allow us to determine the relative importance of these different barriers, nor was it able to identify how providers would prioritize strategies to overcome them. Such information is needed to inform the design of effective, provider-supported quality improvement interventions for diabetes care in First Nations communities.

### Phase II

#### Study Participants

Of the 19 partnering communities 4 were non-isolated, 6 were semi-isolated, and 9 were isolated, with populations ranging 500 to 10,000 people. A total of two hundred and forty-four (244) healthcare providers completed the survey, with nurses (n = 123) making up the largest percentage (50%) of this sample (Table 1). The majority of healthcare providers were female (84%) and average length of employment in an Aboriginal community was 11.4 years with an average of 8.9 years in the specific partnering community.

**Table 1:** Characteristics of Survey Participants

	Non-isolated	Semi-Isolated	Isolated	Total
**Total Number of Participating Communities**	**4**	**6**	**9**	**19**

**Total Number of Participating Healthcare Professionals**	**86**	**61**	**97**	**244**

Nurse	44	30	49	123

Physician	15	10	21	46

Community Health Representative	12	8	15	35

Clinic Manager	11	9	9	29

Nutritionist/dietician	2	2	2	6

Diabetes Educator	2	1	1	4

Other (missing job title)	0	1	0	1

#### Factor Analysis

All fifteen items of the survey correlated fairly well and none of the correlation coefficients were particularly large, so no questions were eliminated. The Kaiser-Meyer-Olkin measure of sampling adequacy was 0.91, above the recommend value of 0.5 which confirms that the data were factorable. Bartlett's test of sphericity was significant [*χ*^2^(105) = 1600.8, p < .001]. The communalities were all above 0.4 confirming that each item shared some common variance with other items. Principle component analysis was carried out with all fifteen items. The initial eigen values showed that the first factor explained 44.0% of the variance, the second factor 10.2% of the variance and the third 7.1% of the variance. A varimax with Kaiser normalization rotation was performed and resulting factor loadings are shown in Table 2. Results confirmed that providers perceive the strategies as falling into three of the four factors identified in the qualitative barriers study, namely patient, provider, and systemic factors (Table 3). The following items did not load on the expected factors: (1) "Address staff shortages" was grouped with patient factors; (2) "Address communication difficulties between healthcare staff and patients" was grouped with systemic factors; and (3) "Advocate making diabetes care a priority for clinic staff" loaded equally heavy on provider and systemic factors.

**Table 2:** Factor Loadings (Rotated*) Principal Components

Survey Item	Factor 1	Factor 2	Factor 3
Increase provider knowledge about diabetes management	0.176	0.810	0.105

Improve clinical practice guidelines	0.121	0.773	0.163

Provide specialized training to deal with diabetes related complications	0.283	0.734	0.279

Address staffing shortages	0.669	0.269	0.251

Help patients seek preventative care so they don't only seek acute care	0.721	0.276	0.260

Reduce frequency of missed appointments	0.777	0.237	0.077

Improve adherence to medication	0.842	0.211	0.049

Address environmental factors (e.g. type of food in store) that make it difficult for patients to adopt healthy lifestyles	0.644	- 0.033	0.411

Motivate patients to adopt healthy lifestyles	0.512	0.104	0.445

Address communication difficulties between healthcare staff and patients	0.268	0.369	0.512

Develop a system or improve the system for diabetes management and follow up	0.317	0.573	0.379

Extend the time healthcare staff are in the community	0.120	0.297	0.718

Ease access to post-clinical services (lab, referrals, medications)	0.231	0.321	0.566

Advocate making diabetes care a priority for clinic staff	0.169	0.593	0.563

Advocate making diabetes care a priority for tribal leadership	0.172	0.075	0.748

* Rotation method used was the Varimax with Kaiser Normalization

**Table 3:** Reliability Analysis of the Survey Items Composing the Three Factors

Factors	Survey Items	Cronbach's Alpha	Corrected item-Total correlation	Cronbach's Alpha if item Deleted
*Patient*	Improve adherence to medication	0.848	0.712	0.808

*Patient*	Reduce frequency of missed appointments	0.848	0.643	0.820

*Patient*	Help patients seek preventative care so they don't only seek acute care	0.848	0.712	0.811

*Patient*	Address staffing shortages	0.848	0.646	0.825

*Patient*	Address environmental factors (e.g. type of food in store) that make it difficult for patients to adopt healthy lifestyles	0.848	0.601	0.829

*Patient*	Motivate patients to adopt healthy lifestyles	0.848	0.531	0.841

*Provider*	Increase provider knowledge about diabetes management	0.852	0.682	0.817

*Provider*	Improve clinical practice guidelines	0.852	0.615	0.837

*Provider*	Provide specialized training to deal with diabetes related complications	0.852	0.733	0.806

*Provider*	Advocate making diabetes care a priority for clinic staff	0.852	0.686	0.815

*Provider*	Develop a system or improve the system for diabetes management and follow up	0.852	0.621	0.832

*Systemic*	Advocate making diabetes care a priority for tribal leadership	0.732	0.408	0.733

*Systemic*	Extend the time healthcare staff are in the community	0.732	0.591	0.630

*Systemic*	Ease access to post-clinical services (lab, referrals, medications)	0.732	0.568	0.645

*Systemic*	Address communication difficulties between healthcare staff and patients	0.732	0.543	0.667

Item analysis was performed for each of the three factors to examine the level of internal consistency and item total correlation. The Cronbach's alphas were 0.848 for patient factors (6 items), 0.852 for provider factors (5 items) and 0.732 for systemic factors (4 items). Eliminating "Address staff shortages" from patient factors; and "Advocate making diabetes care a priority for clinic staff" from provider factors didn't result substantial decrease in alpha, 0.825 and 0.815 respectively. However removing "Address communication difficulties between healthcare staff and patients" from systemic factors reduced alpha from 0.732 to 0.667.

#### Ranking of Strategies

Ranking of the three groupings demonstrated that providers perceive patient factors to be the most important, followed by provider factors and then systemic factors. Table 4 shows the frequency and proportion of responses by impact category. As stated above, analyses were limited to responses of "large impact"; thus the percentages presented in the following sections are based the number of "large impact" responses.

**Table 4:** Frequency and proportion of survey responses by category

Survey Item	Large impact	Some impact	Small impact	No impact	Don't know	No Answer	Total
Increase provider knowledge about diabetes management, n(%)	130(53.3)	74(30.3)	23(9.4)	13(5.4)	3(1.2)	1(0.4)	244(100)

Improve clinical practice guidelines, n(%)	92(37.7)	82(33.6)	35(14.3)	23(9.4)	8(3.3)	4(1.6)	244(100)

Provide specialized training to deal with diabetes related complications, n(%)	127(52.0)	84(34.4)	19(7.8)	5(2.0)	5(2.0)	4(1.6)	244(100)

Address staffing shortages, n(%)	149(61.1)	50(20.5)	14(5.7)	17(7.0)	11(4.5)	3(1.2)	244(100)

Help patients seek preventative care so they don't only seek acute care, n(%)	175(71.7)	48(198.7)	13(5.3)	2(0.8)	4(1.6)	2(0.8)	244(100)

Reduce frequency of missed appointments, n(%)	144(59.0)	67(27.5)	19(7.8)	4(1.6)	9(3.7)	1(0.4)	244(100)

Improve adherence to medication, n(%)	166(68.0)	56(23.0)	11(4.5)	2(0.8)	7(2.9)	2(0.8)	244(100)

Address environmental factors (e.g. type of food in store) that make it difficult for patients to adopt healthy lifestyles, n(%)	149(61.1)	58(23.8)	21(8.6)	6(2.5)	8(3.3)	2(0.8)	244(100)

Motivate patients to adopt healthy lifestyles, n(%)	184(75.4)	43(17.6)	12(4.9)	2(0.8)	2(0.8)	1(0.4)	244(100)

Address communication difficulties between healthcare staff and patients, n(%)	95(38.9)	87(35.7)	44(18.0)	8(3.3)	8(3.3)	2(0.8)	244(100)

Develop a system or improve the system for diabetes management and follow up, n(%)	123(50.4)	84(34.4)	20(8.2)	10(4.1)	4(1.6)	3(1.2)	244(100)

Extend the time healthcare staff are in the community, n(%)	85(34.8)	73(29.9)	33(13.5)	32(13.1)	18(7.4)	3(1.2)	244(100)

Ease access to post-clinical services (lab, referrals, medications), n(%)	86(35.2)	71(29.1)	30(12.3)	39(14.8)	14(5.7)	4(1.6)	244(100)

Advocate making diabetes care a priority for clinic staff, n(%)	99(40.6)	77(31.6)	40(16.4)	20(8.2)	4(1.6)	4(1.6)	244(100)

Advocate making diabetes care a priority for tribal leadership, n(%)	145(59.4)	50(20.5)	29(11.9)	7(2.9)	11(4.5)	2(0.8)	244(100)

##### Patient Factors

The top three ranked strategies were all patient-related: (1) "Motivate patients to adopt healthy lifestyles", (2) "Help patients seek preventative care", and (3) "Improve adherence to medication," with 75%, 72%, and 68% of participants selecting these factors as having the largest impact on diabetes care, respectively.

##### Provider Factors

Strategies pertaining to providers were ranked considerably lower than patient-related factors. The highest ranking clinical strategies, at 53%, were "Increase provider knowledge about diabetes management" and "Provide specialized training to deal with diabetes related complications." Only 41% of providers ranked "Advocate making diabetes care a priority for clinic staff" as having a large impact.

##### Systemic Factors

Factors relating to health system design were least often cited as having a "large impact" on diabetes care. For example, only 35% of respondents ranked "Ease access to post-clinical services (lab, referrals, medications, etc)" and "Extend the time healthcare staff are in the community" as having a "large impact."

#### Variation by Isolation Level

The top three strategies across all communities were patient-related. Since the strategy "address staff shortages" was also grouped within the patient factor, this remained true when the strategies were analyzed by isolation level. Table 5 below demonstrates some variation in the type and order of the different patient strategies by isolation level.

**Table 5:** Top Ranking Strategies by Isolation Level

Ranking	Isolated	Semi-Isolated	Non-Isolated
1	Motivate patients to adopt healthy lifestyles (73%)	Motivate patients to adopt healthy lifestyles (80%)	Motivate patients to adopt healthy lifestyles (76%)

2	Help patients seek preventative care so that they don't only seek acute care (71%)	Help patients seek preventative care so that they don't only seek acute care (77%)	Improve adherence to medications (74%)

3	Address environmental factors that make it difficult for patients to adopt healthy lifestyles (68%)	Address staff shortages (70%)	Help patients seek preventative care so that they don't only seek acute care (71%)

Analysis by isolation level also found that "Ease access to post-clinical services (lab, referrals, medications)" varied significantly between non-isolated (49%) and isolated (23%) communities (*χ*^2 ^= 12.7, *p *< .001).

#### Variation by Healthcare Provider Type

Some variation in participant responses was associated with being a doctor, nurse, or CHR. However, being a clinic manager, diabetes education, nutritionist or dietician did not seem to affect participant responses. Doctors and nurses were more likely to rank patient strategies as key to improving diabetes care than CHRs: 84% of doctors ranked "Motivate patients to adopt a healthy lifestyle" as a strategy with "large impact" compared to 60% of CHRs (*χ*^2^= 5.797, *p *< .05); 65.9% of nurses ranked "Reduce missed appointments" as having a "large impact" compared to only 37.1% of CHRs (*χ*^2 ^= 9.320, *p *< .01). Despite a focus on patient factors, only 25.6% of doctors ranked "Address communication difficulties between healthcare staff and patients" as having a "large impact" compared to 51.4% of CHRs. (*χ*^2 ^= 5.519, *p *< .05)

Providers varied in their ranking of the need for specialized training: CHRs (65.7%) and nurses (59.8%) both signaled greater need for specialized training than did doctors (16.3%). The same trend was seen with the strategy "Increase provider knowledge about diabetes management" and "Improve clinical practice guidelines." Nurses also ranked clinic management strategies as having a significantly higher impact than did doctors. For instance, 49.2% of nurses ranked "make diabetes a priority for clinic staff" as having a large impact compared with 7% of doctors (*χ*^2 ^= 23.9, *p *< .01). Further, 58% nurses ranked "Develop a system for diabetes follow-up" as having a large impact compared with only 30% of doctors (*χ*^2 ^= 9.6, *p *< .01).

## Discussion

Aboriginal communities in developed countries are facing an epidemic of diabetes, but providers working in these environments are often faced with many challenges beyond those experienced in non-Aboriginal settings [14]. This two-phase study revealed that healthcare professionals group the barriers to providing diabetes care in First Nations communities into patient, provider and system-level factors, and they perceive strategies that address patient factors to be the most important to improve quality of diabetes care.

### Ranking of Strategies

Addressing patient factors such as changing lifestyle, seeking preventive care and improving medication adherence was considered the most important approach to improving diabetes care. While one has to be careful to interpret provider *perceptions *of the best strategies with those that will actually impact care, this finding suggests that providers will be supportive of patient-focussed interventions. However, providers' support for patient strategies may also reflect a desire to blame patients for challenges associated with diabetes management in First Nation communities. A study conducted by Sonnenberg of gastroenterologists suggests that providers sometimes resort to blaming their patients for their medical conditions rather that admitting to the failures of disease management, thus making patient blaming an easy "exit strategy" for challenging or complex medical conditions [26]. Sonnenberg notes that this strategy is viewed as being the most productive (highest expected outcome for the physician) for single-visit interactions with patients. However, when patients are seen on a regular basis it becomes more obvious that cooperation and co-management is more productive. Since many rural and remote First Nation communities experience a high turnover of staff, it is possible that this clinical context enables a culture of patient blaming [27].

The impact of physician attitudes and perceptions on their management of diabetes patients has been noted elsewhere in the literature [28-30]. In one study, physicians reported that: "patients do not have a sense of urgency or understanding about treating their diabetes and thus are less likely to adhere to provider recommendations" [28]. This perception diminishes the knowledge and perspective of patients and suggests that providers do not (or could not) play a role in patient education. It is clear elsewhere in the literature, however, that health outcomes for patients with diabetes are improved when empowered patients work with informed providers [28,31].

The lack of support for strategies within the provider factor -- the strategies over which providers potentially have the most control -- suggests that providers may not be aware of the potential impact that quality improvement initiatives can have on diabetes care. Successful initiatives from the United States [4,32] and Australia [33] can serve as useful teaching points and models for the development of local, regional, and national quality improvement programs.

### Variation by Isolation

The communities involved in the CIRCLE study were distributed across isolation levels, with only slightly more isolated communities than non- and semi-isolated communities. We hypothesized that the degree of isolation would have a significant impact on provider responses. While isolation level did have an impact, it was not as strong or consistent as was expected and the association was sometimes counterintuitive. Two results were particularly surprising. First, "address staff shortages," which was grouped under the patient factor, was ranked as the third most important strategy by semi-isolated communities, but did not factor in the top three for isolated communities (which are generally remote, fly-in communities). The reasons for this may be due to differences in the recruitment and retention systems for isolated and semi-isolated communities. Isolated communities tend to attract and retain staff using subsidies, attractive salary packages, and ample vacation time [34]. Semi-isolated communities may not have as attractive compensation packages compared to isolated ones.

Second, non-isolated communities were significantly more concerned with access to post-clinical services (49%) than isolated communities (23%). This is surprising given the remote location of isolated communities. Providers working in isolated communities generally provide a wide range of services to community members, as access to off-reserve care is often expensive and limited. Providers working in semi- and non-isolated communities, on the other hand, have a more diffuse population that may access services through other providers. Services are also more diffuse, meaning that providers rely more heavily on referrals. This combination of factors may actually make accessing consistent services in semi- and non-isolated communities more difficult and may make follow-up with post-clinical services less coordinated than in places with only one entry point for care.

### Variation by Provider Type

Analysis by provider type found some significant differences in perceptions of important diabetes strategies among doctors, nurses and CHRs. There was no significant variation in responses of providers who were classified as clinic mangers, diabetes educations, and dieticians/nutritionists. For diabetes educators and dieticians/nutritionists this is likely due to the small sample size, with only 6 and 4 respondents respectively. It was more surprising that the 29 clinic managers did not significantly differ from nurses, doctors, and CHRs. It is possible that clinic managers group with the provider type reflective of their training (i.e. grouping with nurses if they are a registered nurse or physicians if they are an MD) or that their voices were diffuse and did not group cohesively.

The variations between the responses of doctors, nurses, and CHRs are interesting and require further discussion. For instance, being a CHR or a doctor affected respondents' perceptions of the importance of communication: CHRs viewed communication between patients and providers as significantly more important than doctors. This finding is particularly interesting given providers' strong overall focus on patient strategies and some of the concerns raised previously in this section about the potential influence of patient blaming by providers. The differences identified between CHRs and other providers is worth considering further as CHRs are generally Aboriginal and from the community and, thus, potentially more likely to have similar life experiences or be more empathetic to the perspectives and needs of patients than to the culture and background of physicians and nurses (who, for the most part, are not Aboriginal) [35,36].

The literature on patient-provider communication links good communication with improved patient health outcomes [37] and notes that barriers to effective patient-provider education stem from social and cultural differences between patients and providers. These include: lack of provider understanding of the history of Aboriginal people and the negative impacts of this history on life today (i.e. poverty, poor housing, high unemployment, and expensive food in the north); limited time for patient-provider dialogue; and, cultural differences in verbal and non-verbal communication [38-41]. Teaching healthcare providers effective communication skills and educating them about the history of Aboriginal people (including the negative impacts of colonization and the residential school system), the importance of trust-building, and how to make time for effective communication has the potential to improve provider communication with Aboriginal patients, which may positively influence patient outcomes [41]. However, the literature also notes that changing provider behaviour is difficult: in one study, the participation of doctors in intensive communication skill training did not improve their adherence to communication best practice [42]. Overcoming the underlying patterns of social distancing (and sometimes 'Othering') that stems from unprepared providers working in remote communities with social conditions different from those normally experienced by healthcare providers is another significant challenge [43].

An alternative strategy is to focus on enhancing the role of community health representatives. Their unique position in the community, along with their desire for more specialized training, suggests that with greater support CHRs could serve as a much-needed bridge between the community and the clinic.

### Strengths and Limitations

Developing surveys from rich qualitative work is an effective way to gain a holistic perspective of a particular issue in health research [44]. This study adopted this approach by building a survey of strategies for improving diabetes care in First Nations communities from a qualitative study in which providers identified three thematic categories of the different barriers to care. This well-grounded survey provided interesting and relevant results about provider perceptions of the relative importance of different strategies and variation of provider responses across isolation level and provider type. To our knowledge, this is the first survey to include a national sample of healthcare providers working in 19 geographically and culturally diverse First Nations communities in Canada.

There were also some limitations with this design. The first concern is the representativeness of the sample: the CIRCLE study was based on a sampling frame where communities were recruited based on pre-existing relationships with researchers. While this may have caused some bias in the results, the lack of heterogeneity of the responses suggests that it was not a major concern; key issues seemed to be common across communities. Secondly, the heavier weighting of nurses and doctors in the sample meant that the results likely reflected the views of these two types of healthcare provider types. Comparisons enabled us to reveal the statistically significant differences between different provider types (i.e. doctors versus CHRs) and, thus, reflect the heterogeneity within our sample. Because of the small number of some provider types (i.e. dieticians and diabetes educators), no significant comparisons could be made. Finally, the CIRCLE study focussed on assessing the quality of diabetes care in First Nations communities. While this clinical focus was important, it did not permit the inclusion of a patient voice. We believe that the viewpoint of patients and how they perceive the current context of care is absolutely essential to complete the picture.

## Conclusions

The results of this survey have important implications for practice, policy, and research.

### Practice

Robust findings show that patient factors are an issue of great importance to providers, which suggests that providers may be receptive to quality improvement strategies and chronic care programs focused on patient engagement. One concern with providers' focus on patient factors is that it may be a guise for patient blaming or for off-loading responsibility. However, providers can play a role in patient engagement, such as understanding patient perspectives, local history and culture, culturally appropriate self-management support programs, and broader quality improvement initiatives. Such initiatives could include clinic reorganization with increased training of CHRs so that they can offer peer-counseling in diabetes management, and help entire families or indeed the entire community to adopt healthier lifestyles [45-47]. Changes such as this may help refocus providers on their own role in diabetes management.

### Policy

Policy-makers play a key role in the implementation of research into practice. This study identified two particular areas where the knowledge and resources of policy-makers could facilitate greater understanding and potential solutions to identified challenges: (1) Staff Shortages - policy-makers should investigate staffing levels in semi-isolated communities and develop solutions to overcome potential human resource challenges in these communities; (2) CHR Training - policy-makers should explore the potential for further CHR training and support.

### Research

This study focused on provider perspectives to diabetes care in an effort to better inform quality improvement interventions. Research incorporating patients' perspectives about factors that hinder or improve diabetes care is absolutely needed to complete this picture. Comparing these viewpoints may also help facilitate deeper conversations and analyses about patient-provider communication and the roles of patients, families and communities, as well as their providers in diabetes treatment and management.

## Competing interests

The authors declare that they have no competing interests.

## Authors' contributions

All authors had access to study data and had final responsibility for the decision to submit for publication. All authors contributed to study design, data collection and data interpretation, and all authors revised and approved the report.

## Acknowledgements and funding

This article was written on behalf of the CIRCLE Study Group.

The authors would like to acknowledge all the site investigators, Aboriginal communities, Band Councils and community research assistants who participated in the CIRCLE study.

***British Columbia***, Site Investigator: Dr. Keith Dawson. Research Assistants: Sue Gladstone, Laurel deGoeij, Kelly Legere.

***Alberta***, Site Investigator: Dr. Ellen Toth. Research Assistants: Natalie White Quills, Darci Healy, Theresa Campiou, Brenda Laboucan, Pam Cooke, Daniel Wildcat.

***Saskatchewan***, Site Investigator: Dr. Roland Dyck. Research Assistants: Wendy McKenzie, Teresa McLeod.

***Manitoba***, Site Investigator: Dr. Nichole Riese. Research Assistants: George Flett, Cheyanne Harper.

***Ontario***, Site Investigators: Dr. Bernard Zinman, Dr. Onil Bhattacharyya, Dr. Michael Green, Dr. Anthony Hanley. Coordinating Centre: Dr. Stewart Harris, Mariam Naqshbandi, Jim Esler, Marnie Orcutt. Research Assistants: Elizabeth Estey, Peggy Sugarhead, Denise Troutlake, Linda Nakogee, Tom McLeod, Tina McLeod, Sharon Dockstater, Vikki Tran, Laverne Fiddler, Marie-Elaine Delvin.

***Quebec***, Site Investigators: Dr. David Dannenbaum, Ms. Joceline Piché, Dr. Darlene Kitty, Dr. Laura MacLaren, Dr Ann C. Macaulay. Research Assistants: Jean-Pierre Desormiers, Cindy Chakapash, Lisa Bobbish, Dawn Montour, Cynthia Deere.

***Newfoundland and Labrador***, Site Investigator: Ms. Kayla Collins. Research Assistant: Maggie Organ

Funding for the CIRCLE study was generously provided in the form of a contribution agreement from the Aboriginal Diabetes Initiative (ADI), First Nations and Inuit Health Branch (FNIHB), Health Canada. Irit Rasooly's work was supported by a Fulbright Student Award from the Institute of International Education.

## Pre-publication history

The pre-publication history for this paper can be accessed here:

http://www.biomedcentral.com/1472-6963/11/283/prepub
